# Supplementary material for: Exploring sexual contact networks by analyzing a nationwide commercial-sex review website
Source: PLoS One. 2022 Nov 3;17(11):e0276981. doi: 10.1371/journal.pone.0276981 (PMC9632804; doi:10.1371/journal.pone.0276981)
Supplement: S1 Fig — (DOCX) [file pone.0276981.s002.docx]

S1 Fig. The total number of reviews for each month.
