# Supplementary material for: Exploring sexual contact networks by analyzing a nationwide commercial-sex review website
Source: PLoS One. 2022 Nov 3;17(11):e0276981. doi: 10.1371/journal.pone.0276981 (PMC9632804; doi:10.1371/journal.pone.0276981)
Supplement: S2 Fig — Plot of the average degree of the neighborhood of degree k nodes as a function of k. The green marks correspond to the observed networks, whereas the purple ones correspond to the randomized network preserving the degree distribution. (A) and (B) show the average degree of neighbors of degree k nodes for FCSWs and MCs, respectively. The assortativity coefficient is -0.181. This negative correlation indicates that popular FCSWs are shared by many infrequent MCs. (C) and (D) show the same analysis for the FCSW–FCSW and MC–MC projection networks (right panel in Fig 2A). The assortativity coefficients were positive, suggesting that active MCs tend to review common FCSWs, and popular FCSWs tend to be reviewed by common MCs. (DOCX) [file pone.0276981.s003.docx]

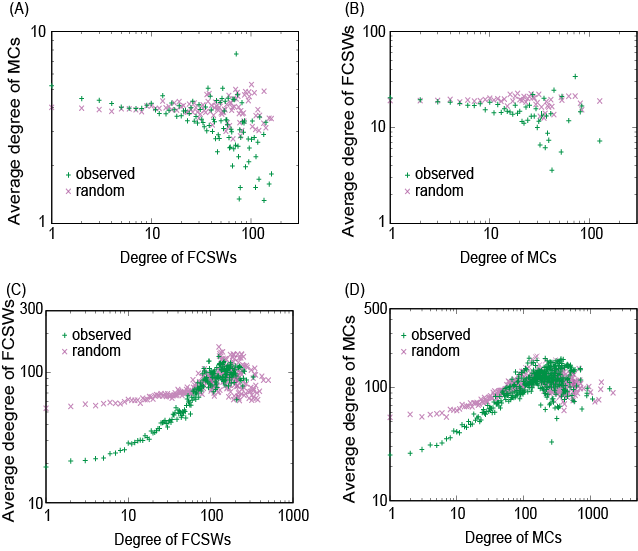


S2 Fig. Degree correlation curve. Plot of the average degree of the neighborhood of degree *k* nodes as a function of *k*. The green marks correspond to the observed networks, whereas the purple ones correspond to the randomized network preserving the degree distribution. (A) and (B) show the average degree of neighbors of degree $k$ nodes for FCSWs and MCs, respectively. The assortativity coefficient is -0.181. This negative correlation indicates that popular FCSWs are shared by many infrequent MCs. (C) and (D) show the same analysis for the FCSW–FCSW and MC–MC projection networks (right panel in Fig 2A). The assortativity coefficients were positive, suggesting that active MCs tend to review common FCSWs, and popular FCSWs tend to be reviewed by common MCs.
