# Supplementary material for: Exploring sexual contact networks by analyzing a nationwide commercial-sex review website
Source: PLoS One. 2022 Nov 3;17(11):e0276981. doi: 10.1371/journal.pone.0276981 (PMC9632804; doi:10.1371/journal.pone.0276981)
Supplement: S1 Table — (DOCX) [file pone.0276981.s004.docx]

**S1 Table**. Model selection for degree distribution

|  | FCSWs | | MCs | |
| --- | --- | --- | --- | --- |
|  | BIC | AIC | BIC | AIC |
| Power-law distribution | 82818 | 82733 | 106699 | 106645 |
| negative binomial distribution | 82895 | 82871 | 111421 | 111395 |
| log-normal distribution | 85108 | 85092 | 115047 | 115029 |
