# Supplementary material for: Exploring sexual contact networks by analyzing a nationwide commercial-sex review website
Source: PLoS One. 2022 Nov 3;17(11):e0276981. doi: 10.1371/journal.pone.0276981 (PMC9632804; doi:10.1371/journal.pone.0276981)
Supplement: S1 Appendix — (DOCX) [file pone.0276981.s005.docx]

**S1 Appendix. Soaplands in Japan**

In Japan, soaplands are official bathhouses where a male customer (MC) can have sexual intercourse with a female staff member who is the sole proprietor entrusted to wash the MC’s body [S1]. By legal definition, soaplands “establish a private room as a facility for bathhouse business and provide services to contact clients of the opposite sex in the private room.” This type of establishment was already common in the 1960s. In Japan, prostitution is prohibited by the Prostitution Prevention Law enacted in 1956 although there is no punishment for prostitution itself, that is, no punishment for MCs and female commercial sex workers (FCSWs). Soaplands have developed as a way to circumvent this law. In reality, soaplands are regarded as the only licensed facilities in Japan that offer vaginal intercourse; in other sex establishments, only non-vaginal intercourse, such as oral and anal sex, is allowed to be offered because an act is classified as prostitution in Japan only when vaginal intercourse is involved. According to a report by the National Police Agency in 2021, there were 1,185 soaplands in Japan [S2]. In the days before the development of the Internet, it was usually necessary to visit establishments to obtain accurate information about FCSWs working there. Nowadays, it is possible to obtain information on adult websites, such as the target website. An MC can choose an FCSW by paying reservation or selection fee or leave the choice to the establishment without additional fee. Due to the language barrier and work visa issues, most of MCs and FCSWs are Japanese or permanent residents of Japan. After peaking in 1984, the number of soaplands has tended to decline, but for the past quarter century, the number has remained around 1,200.

Annual change in number of licensed soaplands in Japan [S2].

[S1] Aoyama K, The sex industry in Japan: The danger of invisibility. In: McLelland M, Mackie V, editors. Routledge Handbook of Sexuality Studies in East Asia. Oxon: Routledge; 2015.

[S2] National Police Agency, White Paper on Police. Available from: https://www.npa.go.jp/publications/whitepaper/index_keisatsu.html.
