# Supplementary material for: Exploring sexual contact networks by analyzing a nationwide commercial-sex review website
Source: PLoS One. 2022 Nov 3;17(11):e0276981. doi: 10.1371/journal.pone.0276981 (PMC9632804; doi:10.1371/journal.pone.0276981)
Supplement: Attachment — Submitted filename: Response to Reviewers.docx [file pone.0276981.s006.docx]

**POINT-BY-POINT RESPONSE TO REVIEWERS**

Dear Reviewers,

We are grateful for the time and effort you have dedicated to providing insightful feedback on ways to strengthen our manuscript. We have carefully considered the comments and thoroughly revised the paper. In this response letter, your comments and advice are highlighted in yellow. Point-by-point responses to the comments and suggestions are shown in bold italic font. Red text indicates the addition of the text to the revised manuscript.

Reviewer #1: The study attempts to study networks of sexual contacts in Japan derived from online reviews.

The manuscript presents an interesting data set and some interesting results however misses to provide robust findings and discuss implications of the network analysis to public health, or sociology, or economy, limiting itself to a network data analysis study.

***We would like to thank the esteemed reviewer for the insightful remark. We agree with you and acknowledge that this study is important for public health, including the spread of STIs. Our original motivation for the current study was to elucidate the characteristic of the social network of sexual contact in public health perspectives (Ito et al. 2019 Appl Math Comput 348: 363-370, Ito et al. 2019 Scientific Reports. 9: 17408, 2019, and Ito et al. 2021 Theor Biol Med Model 18: 18, 2021). However, we had realized that we should not give the impression to the readers that soaplands (and the sex industry) are a possible reservoir of STIs. Therefore, while we recognized and partially described the importance of discussing public health applications, we could not ignore the sex workers and establishments that may be offended or injured by this study. For these reasons,*** ***we admit that our initial manuscript was half-hearted: discussing STIs but lacking epidemiological analysis, as you critically mentioned.***

***Recently, our interest shifted from the public health issue of STIs to investigating the sexual contact network itself from social science and anthropological perspectives (Ito et al. 2019 PLOS ONE e0221520, 2019). This is because we found during the research process that there are few academic studies that have quantitatively investigated what is taking place in the Japanese sex industry, and that the actual situation has been shrouded in mystery. Therefore, I would like to emphasize that this study is the first one that deals with the actual situation of the Japanese sex industry (especially soaplands) from a mathematical scientific viewpoint and that it is a mathematical analysis of sexual contact networks from a social scientific viewpoint.***

***Since our analysis does not provide evidence that the sex industry is a hotspot of STIs, we have been careful to avoid creating a prejudice against sex workers or the sex industry regarding STIs. Thus, we do not extend our discussion to STI prevention policy in this version. In addition, to maintain legality, the target website is organized under the public stance that prostitution does not take place in soaplands, and thus an MC cannot write*** ***directly about sexual intercourse in his review (see detailed information about soaplands in S1 Appendix in Supporting information). Therefore, due to our respect for the public stance of the website and business form of soaplands, we have kept the discussion of the application to STIs and public health to a minimum. Since this study uses only publicly available online information, it does not involve issues of medical ethics, as assured by our institution's ethics committee. However, there are sensitive aspects of this study that could lead to a threat to the interests of popular FCSWs and establishments by insisting on a relationship between them and STIs.***

***We have carefully rewritten the entire document including Abstract and Author Summary to avoid prejudice and misunderstanding regarding its impact on STIs and public health and to clarify the limitations of this study.***

Furthermore, it needs a thorough review to improve the language, explanations, remove redundant information, and clarify the purpose and meaning of some measures.

***I would like to thank you for this feedback. We agree with you and apologize for our unclear explanations. We have carefully updated our explanations, as will be described in detail below.***

It also needs a more proper contextualisation with the literature given that several studies have addressed topics discussed in the manuscript, such as Internet-mediated prostitution and epidemic spreading.

***Thank you for pointing this out. We have scrutinized the previous studies and added some citations in addition to the paper referred to by you.***

I cannot recommend the current version of the manuscript for publication on PLoS ONE or any other journal but I encourage the authors to address the comments below and revise the manuscript accordingly.

***Thank you for giving us the opportunity to revise our manuscript. We have revised carefully as follows.***

- The meaning/scope of Prefecture should be explained, given that this definition is not much used in most countries.

***Thank you for pointing this out. As the term "prefecture" is unfamiliar in many countries, in Author Summary, “prefectures” has been replaced with “locations.” In addition, we have added explanation about prefectures as follows:***

P4, L83-84. prefecture (the largest administrative division in Japan, see Fig 1A for the spatial division)

P4, L84 - P5, L85. Soaplands are licensed by ordinances of each prefecture and are monitored by the prefecture police.

- An explanation of "soapland" should be given already in the introduction, e.g. the text in the methods could be more concise and merged in the introduction.

***We would like to thank you for the great suggestion. Soaplands are a unique Japanese business practice, where prostitution, which should be illegal in Japan, is legally conducted. It is difficult for even us Japanese to understand why this type of business system is allowed (using loopholes in the law). Briefly, soaplands are licensed brothels, which have existed since the 1960s.*** ***Since the historical background and legal interpretation of the soapland are not relevant to the subject of the paper, we have moved these details to the Supporting information. We have also given a brief description of soaplands in the Introduction:***

P4, L67-76. We constructed a review network between MCs and FCSWs and analyzed its properties, focusing only on FCSWs working in Japanese brothels called “soaplands” that have existed since the 1960s. Sex establishments other than soaplands are prohibited from offering sexual intercourse in exchange for money (for more information on soaplands, see S1 Appendix). FCSWs can work legally at a licensed soapland, and they can engage in sex work while being protected from violence and various problems caused by the MCs by the soapland to which they belong, unlike street sex workers.

In 2021, there were 1,185 soaplands regulated by the National Police Agency. Soaplands were scattered throughout Japan, and the study website covered 66% of them (Fig 1A).

- Female commercial sex workers -> Female sex workers

***We discussed at length which expression we should use here. We decided to label it “commercial” because female employees of soaplands are not only sex workers but are also engaged in the commercial sex industry in Japan. Moreover, “commercial sex worker” is a frequently used term [20,21].***

- p4, l61-65. The authors should write these points using a more public-health language, i.e. that sex-workers are a risk group or at risk group, that is more exposed to potential STIs in case of unprotected sex, etc... similarly to clients.

***We would like to thank you for the suggestion. In the revised manuscript, we have omitted the public health content from the first paragraph of the Introduction and have only included the following text:***

P3, L59 - P4, L62. While it has traditionally been extremely difficult to obtain information on “who had sexual contact with whom,” recently, studies have been conducted using sex industry websites that publish information that provides a basis for assuming that sexual contact has occurred [11–16].

- The 3 types of random models should be better explained. What do authors mean by "randomly rewired networks without changing the XX"? I assume that some network properties are being conserved but it is not clear.

***We would like to thank you for the great suggestion. We have added the following text to explain randomly rewired networks in Introduction and Results***

P4, L81-84. We performed network analysis and compared the empirical network to three randomized artificial networks to see the tendency of each MC to limit his contacts with FCSWs to specific prefecture (the largest administrative division in Japan, see Fig 1A for the spatial division) and establishment.

P6, L118-124. In Type I, only the degree distributions of FCSWs and MCs are preserved, and other network structures are destroyed. Type II additionally preserves location information in the form of prefectures. In other words, network properties originating from the tendency of MCs to move between prefectures is conserved. Type III additionally preserves information of establishments. This means that the network properties originating from the tendency of MCs to use certain establishments are conserved.

----> fig 2b should first show the empirical network and then the randomized models

***Thank you for your suggestion. We have changed Fig 2B accordingly.***

- The purpose of fig 1C and fig 1D must be clarified. In principle, they do not bring any relevant insights to understand the follow up analysis. I suggest only showing the "enlarged" part of fig 1d.

----> Similarly, fig 1A could be simplified.

***Thank you for your suggestion. Fig 1C and Fig 1D are schematic visualizations of the entire networks and are not intended to provide scientifically solid findings or insight, as you state. Such diagrams have been frequently used in network science and are useful when making a quick comparison between networks. Further, Fig 1A is useful for readers who are not familiar with the locations and sizes of the prefectures in Japan. We have retained the original figure and added explanatory text.***

P5, L106 - P6, L112. Fig 1B shows weighted links between prefectures, which are defined as the number of MCs who wrote reviews of the FCSWs in the two prefectures. In other words, the thickness of the line between prefectures indicates how often MCs used establishments across prefectures. At the establishment level, the network is connected (Fig 1C), but at the individual level, there are many small, connected components in addition to the giant connected component (Fig 1D and Table 1). The enlarged panel in Fig 1D shows that there are many patterns that link one FCSW to many MCs and vice versa.

- Table 1 should be simplified and only contain relevant information that brings insights.

***Thank you for your feedback. Per your suggestion, we have*** ***made Table 1 as concise as possible by deleting unimportant items and moved the resulting model selection figures for the degree distributions to S1 Table.***

- The bi-partite clustering coefficient should be described since it is not a standard network measure.

***Thank you for your suggestion. We have added an explanation of the bipartite clustering coefficient.***

P8, L149-151. The conventional clustering coefficient is defined as the ratio of 3-cycles and 2-paths, while the bipartite clustering coefficient is defined as the ratio of 4-cycles and 3-paths (see Material and Methods).

- Fig 4 should be removed since it is not much informative, given that assortativity can be measured and conveys the same information.

***Thank you for your suggestion. We have moved Fig 4 to Supporting information as S2 Fig, which is necessary because it allows us to confirm correlations that cannot be seen with r alone, such as in the case of nonlinear correlations.***

- P12, l250. Which year and/or month was the data collected? It corresponds to which period? Part of this is written elsewhere.

***Thank you for your comment. As noted in Materials and Methods, data was scraped on April 4, 2021. The data obtained here consisted of reviews of the FCSWs that were registered on the website on that day. We have added the following sentence and S1 Fig.***

P4, L75-78. We focused on reviews that were posted on the target website and were available to anyone online on April 4, 2021. Here, 96.4% of the reviews on the site at that time were written after the year 2020 (S1 Fig).

- The section "Methods: Spreading on the Network" is misleading because there is no epidemic model being studied in this manuscript. Eq 2 is not necessarily true for other models than SIS and SIR, and the authors should specify what they want to study/capture with this epidemic threshold? It's OK to use this measure but limitations should be clearly described, and the scope in terms of STIs clarified.

***Thank you for your suggestion. We have changed the heading “Spreading on the Network” to “Robustness of the network.” We have added the following explanation about the epidemic model we consider.***

P17, L378-390. For example, in an SIS model, if the probability of infection of individual $i$ is denoted by $\rho_{i}$, β is the infection rate, and γ is the cure rate, we obtain

$$\frac{{d\rho}_{i}}{dt}=-\gamma\rho_{i}+\left( 1-\rho_{i} \right)\beta\sum_{j=1}^{N} A_{ij}\rho_{j}$$

using individual-based mean-field theory [28–31]. For the effective transmission rate $\lambda=\beta/\gamma$, the infection threshold is given as

$$\lambda\geq\lambda_{c}, \lambda_{c}=\frac{1}{\Lambda_{1}}.$$

An exact calculation [32] yields

$$\lambda_{c}\geq\frac{1}{\Lambda_{1}}.$$

The same result was obtained when SIR, SIRS, or SEIR was used instead of SIS [33]. Note that the weights of the links in the network and the temporal dynamics of the contacts are not considered here. Thus, $\Lambda_{1}$ is an indicator of the strength of spread on a simple graph, not an indicator that can predict the final number of infected persons.

- The text in Methods should (in general) be more concise and show the definitions of measures and equations when appropriate (e.g. clustering coefficient for bi-partite network), the randomisation procedure appears in the methods and in the main body of the manuscript (unclear in both cases).

***Thank you for your suggestion. We have improved the description in Method as itemized below.***

- ***We have added an explanatory note about parameter fitting below Eq. (1).***

P15, L338-340. The parameters $\alpha$ and $k_{\min}$ are estimated by model selection using AIC and BIC. Furthermore, the model selection confirmed that the power distribution was better than binomial and lognormal distributions [10,24,25].

***- We have added a definition formula for the clustering coefficients in Materials and Methods:***

P16, L352-353.

$$C_{RA}=\frac{4C_{4}}{L_{3}},$$

where $C_{4}$ is the number of 4-cycles, and $L_{3}$ is the number of 3-paths in a bipartite network.

***-We have rewritten the subsection on degree correlations in Materials and Methods, adding a definition formula:***

P16, L358-362. To quantify the degree of correlation, we calculated the assortativity coefficient, which is the Pearson correlation coefficient of the degree between pairs of linked nodes [27]:

$$r=\frac{\sum_{ij} \left( A_{ij}-\frac{k_{i}k_{j}}{2m} \right)k_{i}k_{j}}{\sum_{ij} \left( k_{i}\delta_{ij}-\frac{k_{i}k_{j}}{2m} \right)k_{i}k_{j}},$$

where $k_{i}$ is the degree of individual $i$, and $m$ is the number of links.

- ***We have added an explanation for the randomization procedure, as mentioned in my response to the comment above.***

P6, L118-124. In Type I, only the degree distributions of FCSWs and MCs are preserved, and other network structures are destroyed. Type II additionally preserves location information in the form of prefectures. In other words, network properties originating from the tendency of MCs to move between prefectures is conserved. Type III additionally preserves information of establishments. This means that the network properties originating from the tendency of MCs to use certain establishments are conserved.

- What are the implications of these findings for public health (or other disciplines)? How do they compare with previous literature?

***As explained in our first response, we have kept the discussion of the application to STIs and public health to a minimum. In Introduction, we have introduced previous studies, including the references you mentioned below, and explained the main differences between our study and the previous studies in Discussion.*** ***In addition, we have added the reference Miyazaki et al. [20] which showed that the incidence of STIs among FCSWs in soaplands was not high.***

- I recommend the authors to apply other strategies to study the robustness of the network, e.g. considering specific prefectures or soapland establishments, or other network measures at the individual level. That would enrich substantially the analysis and could provide several insights.

***Thank you for your interesting suggestion. We agree that the ideas you proposed could provide additional insight. However, the*** ***background and the methods of this study did not directly involve specific establishments or sex workers. This study was designed to investigate the properties of the entire review network on the web.***

- Some relevant papers that would help authors to contextualise their findings:

--- about online prostitution data/reviews & advertisement:

https://link.springer.com/article/10.1007/s42001-021-00156-2

https://www.oxfordhandbooks.com/view/10.1093/oxfordhb/9780199915248.001.0001/oxfordhb-9780199915248-e-17

https://www.oxfordhandbooks.com/view/10.1093/oxfordhb/9780199915248.001.0001/oxfordhb-9780199915248-e-3

--- about epidemic spreading on sexual networks of sex-workers and their clients.

https://journals.plos.org/ploscompbiol/article?id=10.1371/journal.pcbi.1001109

https://pubmed.ncbi.nlm.nih.gov/12650777/

--- about sampling sensitive data

https://academic.oup.com/comnet/article-abstract/9/6/cnab034/6420764

***Thank you for the useful information. We have examined these references carefully and added all but the last paper to the bibliography. They are cited in Introduction and Discussion as references [6, 12-15]*.**

Reviewer #2: Thank you very much for your interesting article. You were scraping data from an online website for soaplands and used information on sex workers and male clients to construct a social network and analyse the properties of this network, also with regard to the spread of a hypothetical STI. The article is well written and easily understandable. I have some minor comments, which in my opinion would strengthen the understanding of the data and underline the contribution of your work to understanding the spread of STIs.

***Thank you very much for your high evaluation of our paper. We are pleased to respond to your insightful comments below.***

- You state that 55% of soaplands are covered by the website. From the Map A in Figure 1, I think that there is some geographical bias with regard to soaplands missing on the website. Could this be due to soaplands of specific regions using a different website? (Or in other terms, how random are soaplands missing on the website?)

***Thank you very much for the valuable feedback. Upon closer examination of the data, we found an error in the number of soaplands not listed on the website because there are special bathhouses where sex work is not conducted. Therefore, we have corrected the figures in Fig 1A. In addition to the site we studied, there are several other similar websites, and some websites provide information only for specific prefectures and areas (e.g., the Tokyo metropolitan area). Since the managers of establishments decide which websites to use for their advertisements, a certain percentage of establishments do not use the current website we studied. Hence, the missing is not random as you suggested. We have added the following text.***

P4, L67-69. We constructed a review network between MCs and FCSWs and analyzed its properties, focusing only on FCSWs working in Japanese brothels called “soaplands” that have existed since the 1960s.

P13, L275-276. We have selected the largest website among those with reliable sources for this study. Whether an establishment is registered on this site is the manager’s decision.

- Are there any estimations on the share of commercial sex contacts outside of soaplands? I.e., what role do soaplands play in commercial sex? Are sexual contacts in soaplands more, equal or less likely to perform safer sex? You state that there is no academic investigation in the discussion section. But is there any information on legal proceedings for illegal sex work?

***Thank you very much for the comment. Licensed sex industry operators other than soaplands are not allowed by “the Prostitution Prevention Law” to offer vaginal sex. In 2021, There were only 426 illegal cases reported to the police*** ***in Japan as a whole. There are both soaplands that require the use of condoms and those that do not, but this is not clearly stated on the target website.*** ***In fact, this website does not even mention that sexual intercourse takes place in soaplands because it could be illegal on the charge of mediating prostitution. We discuss this in the S1 Appendix in Supporting information.***

- A similar question would be, if you could provide some context of the incidences of STIs in Japan. Is there any information where people get infected with STIs? Which role deos commercial sex play as an infection setting compared to other settings (private contacts, injecting drugs, etc.).

***Thank you for your question. We have added the following text and references to this question:***

P10, L237-238. furthermore, a previous study reported that the incidence of STIs among FCSWs in soaplands was relatively low [20].

- Would it not be possible to obtain data for the prefectures Kochi and Tochigi now? I get that it would preferrable to have the data collection happening on the same day for all, but IMHO it would be better to have a more complete dataset from different dates than an incomplete dataset from one day. (somewhat related: you state that Kochigi does not allow soaplands, so would it matter that data was not accessible?)

***Thank you very much for your feedback. There was a mistake in the listing of the prefectures, and we apologize. The following nine prefectures have no soaplands: Aomori, Gunma, Kyoto, Nagano, Nagasaki, Nara, Okayama, Toyama, and Yamagata (not Tochigi). We have corrected this in the manuscript (P14, L297-299). We decided not to conduct additional research on the two prefectures with missing data because new workers are continuously joining the website and old workers are disappearing from it. Moreover, the website we studied is frequently updated in terms of design and layout, and when an FCSW retires, the link between the review and the FCSW is lost.***

- Is there some information on the website how many MCs are registered overall?

***Thank you for your question. We have added the following sentence.***

P12, L257-258. According to the website, the number of members exceeded 2 million in March 2021, but only less than 3% of them wrote reviews.

- Can you do some sensitivity analyses how MCs and FCSWs with no reviews could affect the network properties if they behaved differently than the MCs and FCSWs with reviews?

***Thank you for the valuable suggestion. Unfortunately, we have not conducted any sensitivity analysis. Although there is no supporting data, MCs who have written reviews may be more likely to frequent soaplands than MCs who have never written reviews. In this case, adding MCs who have never written reviews would not have much effect. The problem is that MCs who have written reviews do not always write reviews, but may only write about their favorite FCSWs. We have no idea what percentage of cases this is. The strength of the relationship between the presence of reviews and actual actions is a mystery at the moment. The limitations and future works of this study have been added in the last paragraph of Discussion.***

P12, L256 – P13,263. It should be noted that the contacts revealed by reviews are only a small fraction of the actual commercial sexual contacts. According to the website, the number of members exceeded 2 million in March 2021, but only less than 3% of them wrote reviews. Another important limitation is that MCs can have more than one accounts with their linked mobile phone numbers, and FCSWs can have more than one account if they work in more than one establishments. This is an unavoidable limitation of the study since MCs and FCSWs are anonymized. We did not consider the temporal order of the reviews, but that may be important as shown in [12]. Thus, the network presented here is only a small part of the long history of prostitution in Japan.

- Is there a way to avoid having duplicates of FCSWs if they work in different soaplands by trying to find duplicates by name or provided features (potrait, age, etc.)?

***Thank you for the valuable suggestions. Names are not a clue because they are often changed. It is also difficult to automatically identify FCSWs by other features (potrait, age, etc.). We think it is worth a try as a new research plan, but we did not consider duplicates of FCSWs because we avoided ethical issues by limiting this study to structural analysis of the review network on the web and not inquiring about the people who exist behind it.***
